# Assessment of language lateralization in epilepsy patients using the super-selective Wada test

**DOI:** 10.1007/s00701-024-05957-8

**Published:** 2024-02-10

**Authors:** Kazuo Kakinuma, Shin-ichiro Osawa, Kazuto Katsuse, Hiroaki Hosokawa, Kazushi Ukishiro, Kazutaka Jin, Kuniyasu Niizuma, Teiji Tominaga, Hidenori Endo, Nobukazu Nakasato, Kyoko Suzuki

**Affiliations:** 1 Department of Behavioral Neurology and Cognitive Neuroscience, Tohoku University Graduate School of Medicine, 2-1 Seiryo, Aoba-ku, Sendai, 980-8575 Japan; 2 Department of Neurosurgery, Tohoku University Graduate School of Medicine, Aoba-ku, Sendai, Japan; 3 Department of Neurology, The University of Tokyo Graduate School of Medicine Faculty of Medicine, Bunkyo-ku, Tokyo, Japan; 4 Department of Rehabilitation, National Hospital Organization Sendai Nishitaga Hospital, Taihaku-ku, Sendai, Japan; 5 Department of Epileptology, Tohoku University Graduate School of Medicine, Aoba-ku, Sendai, Japan; 6 Department of Neurosurgical Engineering and Translational Neuroscience, Tohoku University Graduate School of Biomedical Engineering, Aoba-ku, Sendai, Japan; 7 Department of Neurosurgical Engineering and Translational Neuroscience, Tohoku University Graduate School of Medicine, Aoba-ku, Sendai, Japan

**Keywords:** Epilepsy, Epilepsy surgery, Functional mapping, Lateralization, Preoperative planning, Wada test

## Abstract

**Background:**

The classical Wada test (cWada), performed by injecting a short-acting anesthetic through the intracarotid route, helps determine language dominance. In the cWada, adverse effects are observed in 10–30% of trials, hindering accurate assessments. In this study, we assessed the effectiveness of the super-selective Wada test (ssWada), a more selective approach for anesthetic infusion into the middle cerebral artery (MCA).

**Methods:**

We retrospectively examined the data of 17 patients with epilepsy who underwent ssWada via anesthetic injection into one M1 segment of the MCA and at least one contralateral trial.

**Results:**

The ssWada identified 12 patients with left language dominance, 3 with right language dominance, and 2 with bilateral language distribution. Nine trials on the language dominant side resulted in global aphasia for patients with left- or right language dominance. Of the 13 trials conducted on the non-dominant language side, 12 revealed intact language function and one resulted in confusion. Among these, the outcomes of global aphasia or no language impairment were confirmed in the contralateral trials. Among the 22 trials of unilateral M1 injections in patients with unilateral language dominance, 21 (95.5%) showed either global aphasia or no language impairment, indicating language dominance.

**Conclusions:**

The ssWada yields clear results, with a high rate of over 90% in determining the language dominant hemisphere with few side effects.

## Introduction

The Wada test [10–12] remains the gold standard for determining language dominant hemispheres. While functional magnetic resonance imaging has partly replaced it over the years, the Wada test remains useful, especially in cases with non-left dominance or atypical background factors [7, 13]. The classical Wada test (cWada) involves administering anesthetics into the cervical segment of the internal carotid artery, temporally suppressing brain function in the perfused regions. Amobarbital has long been used as an anesthetic for the cWada, leading to its colloquial name, the intracarotid amobarbital procedure. However, in recent years, propofol use has become significantly more prevalent [5, 6, 8, 9] as amobarbital is no longer marketed in various countries.

Theoretically, injecting an anesthetic agent into the dominant hemisphere of patients with unilateralized (clearly left- or right-dominant) language function should result in global aphasia, akin to conditions with significant dysfunction in the dominant hemisphere. Studies involving stroke patients indicate that large lesions, including those in the frontal and temporal lobes, in the dominant hemisphere cause global aphasia at a high rate, particularly in the acute phase [15]. However, administering anesthetics into the internal carotid artery on the dominant side does not always result in global aphasia, even among individuals with unilateral language dominance [2, 10, 11]. The feasibility of determining language dominance through a single trial of the Wada test has long been debated [12, 14], and a consensus on the practical use of the unilateral Wada test remains elusive, possibly owing to this uncertainty. One factor that may decrease the accuracy of the cWada results is the cross-flow through the Circle of Willis, which may transfer some portion of the anesthesia to the other side. Another possible cause is uneven drug distribution within the hemisphere, resulting in inadequate anesthetization of language or other regions, making precise assessment of cognitive function difficult. Regarding drug distribution, unnecessary anesthetizations in other brain regions can occur. For example, over-anesthetizing the thalamus, brainstem, and medial frontal cortex may cause confusion, impaired consciousness, or apathy, impeding accurate cognitive evaluation. Mikuni et al. [6] reported that 19 of 58 (33%) patients administered the cWada experienced side effects that affected task performance, with six (10%) patients experiencing confusion. Szantroch et al. [8] reported that among 122 patients across 240 trials, 10 right cerebral propofol infusions and 10 left cerebral infusions (comprising 8.3% of the total) induced behavioral abnormalities, including apathy, drowsiness, verbal disinhibition, and viscosity. They also reported that 4 of 122 (3.3%) patients underwent the Wada test only unilaterally due to complications. Although one might attribute the high rate of impaired consciousness to propofol use, amobarbital also shows similar complications. Kemp et al. [4] reported that valid cognitive assessments were obtained in only 108 of 141 (76%) Wada test trials, with the remainder requiring retests. These results suggest that 10–30% of cWada trials yielded unsatisfactory results due to complications or adverse effects.

To overcome these shortcomings of the cWada, researchers have attempted more selective infusions of anesthetics in recent years. Fujii et al. [1] suggested that selective anesthetic infusion into the middle cerebral artery (MCA) reduces adverse effects in the Wada test. We established a modified version of the Wada test using super-selective catheterization that involves injecting anesthetics selectively into the target arteries corresponding to the planned resection area [3, 16], named the “super-selective Wada test” (ssWada). As most of the brain areas responsible for language are perfused by the MCA, we believe that the ssWada in the MCA region will help adequately anesthetize language-related areas with minimal effect in adjacent regions, compared with the cWada. The ssWada not only helps reduce discomfort and disturbance of consciousness but also enables a more detailed assessment of cognitive impairments during anesthetization [3]. This study aimed to investigate the effectiveness and accuracy of the ssWada in determining language dominance.

## Methods and materials

### Participants

We retrospectively analyzed epilepsy cases examined using the ssWada at Tohoku University Hospital in Sendai, Japan, between January 2018 and December 2022. All patients had a clinical requirement for accurate language distribution assessment. From serial cases, we included those in which the anesthetic was administered into at least one M1 segment on any side, and at least one trial was performed in the contralateral MCA area. We then identified trials involving the M1 and M2 superior division (M2sup) and the M2 inferior division (M2inf) of the MCA among the extracted cases and excluded other trials.

The study was approved by the Ethics Committee of Tohoku University Graduate School of Medicine (2020–1–083) and was conducted in accordance with the principles of 1964 Declaration of Helsinki and its later amendments. Written informed consent was obtained from all patients to publish their information and images.

### Super-selective Wada test

Digital subtraction angiography was performed to delineate the MCA and identify M1, M2sup, and M2inf. Endovascular neurosurgeons inserted microcatheters at the sites of interest (M1, M2sup, or M2inf). After performing a baseline language assessment, propofol was administered [9] through the microcatheter. Language function was reassessed while the anesthetic effect was active in the selected area. All neurological symptoms generally resolved within 10 min, and we assessed language within 5 min. The exact duration of neurological symptoms in each case is given in the supplementary table appended to our previous paper [3]. Language assessment included sequential speech, reading aloud (both words and sentences), repetition (both words and sentences), listening comprehension, reading comprehension, naming, and writing. In this study, we classified the induced language states into three groups: “global aphasia,” characterized by impairments in speech, comprehension, and repetition; “other aphasia,” involving significant language impairments in some areas but not all; and “no language deficits,” indicating the absence of language deficits in any of these tasks. More detailed procedures and assessments of the ssWada are described in our previous papers [3, 16].

## Results

Of the 33 serial cases examined using the ssWada, 17 met the inclusion criteria. Among these, 12 exhibited left-dominant language, 3 exhibited right-dominant language, and 2 demonstrated language representation on both hemispheres. Among the 50 trials involving 17 cases, no irreversible adverse effects, such as artery dissection, pseudoaneurysm, symptomatic ischemia, or intracranial hemorrhage, were observed.

During one trial in the left M1 and one in the left M2inf, the patients experienced transient confusion, resulting in insufficient language evaluation. No adverse effects that disturbed language assessments occurred in other trials, and no trials produced any irreversible complications.

The results for the individual cases are summarized in Table 1. The case numbers were the same as those in the supplementary table of Kakinuma et al. [3]; Cases S-23, S-24, and S-25 have been added to this study.

**Table 1:** Results of the super-selective Wada test of the middle cerebral artery areas

Case	Age	Sex	Handedness	Dominance	Lt M1	Lt M2sup	Lt M2inf	Rt M1	Rt M2sup	Rt M2inf
S-4	24	F	Rt	Lt	G	-	-	-	-	N
S-7	39	M	Rt	Lt	G	O	-	-	N	-
S-9	58	F	Rt	Lt	G	-	-	N	-	-
S-11	19	F	Rt	Lt	G	-	-	N	-	-
S-15	30	M	Blt	Lt	G	-	-	N	-	N
S-17	22	M	Lt	Lt	G	-	-	N	-	-
S-22	37	M	Rt	Lt	G	-	-	N	-	-
S-23	22	M	Rt	Lt	G	O	-	N	-	-
4	29	F	Rt	Lt	-^*1^	G	O	N	N	-
S-24	40	M	Rt	Lt	-	G	O	N	-	-
1	38	M	Rt	Lt	-	O	O	N	-	N
S-25	28	F	Rt	Lt	-	O	O	N	-	-
S-18	16	F	Rt	Rt	N	-	-	G	-	-
6	46	M	Rt	Rt	I	-	-	-	G	O
5	38	M	Blt^*2^	Rt	N	-	I	-	O	O
7	29	M	Rt	Blt	-	G	N	O	-	-
8	32	F	Rt	Blt	O	-	N	O	O	O

*Blt* bilateral, *F* Female, *G* Global aphasia, *I* Indeterminate due to confusion, *Lt* Left, *M* Male, *M2inf* Inferior division of the M2, *M2sup* Superior division of the M2, *N* No language deficit, *O* Other aphasia, *Rt* Right

^*^1; We excluded the left M1 in Case 4 because language assessment and recordings were missing

^*^2; Patient 5 was trained at an early age to use the right arm as the dominant limb

### Infusion of propofol into the language dominant side

Among patients with left dominance, injecting the anesthetic agent into the left M1 produced global aphasia in all eight trials, while none of the contralateral trials (six M1, one M2sup, and two M2inf) resulted in language deficits. In cases of right dominance, infusion into the right M1 produced global aphasia in one patient, whereas infusion into the left M1 did not induce language impairment. In cases of bilateral dominance, one trial on the left M1 and two on the right M1 resulted in language impairments but not global aphasia.

### Propofol infusion into the language non-dominant side

In cases of left dominance, injecting the anesthetic agent into the right M1 did not produce language deficits in all 10 trials, while all contralateral trials (six M1, five M2sup, and four M2inf) showed significant language impairments. In cases of right dominance, injection of the anesthetic agent into the left M1 demonstrated no language deficits in two of the three cases; in one case, language assessment was challenging owing to confusion. The right dominance in these three cases was confirmed by apparent language symptoms in the contralateral trials (one M1, two M2sup, and two M2inf).

### Availability of unilateral Wada testing

Overall, there were 25 trials involving M1: 22 trials in cases with right or left unilateralized language distribution and 3 in those with bilateral language function. Among the 22 trials involving unilateralized (left or right) distribution, 21 (95.5%) yielded distinct results: either “global aphasia” or “no language impairment.” Furthermore, these 21 results were consistent with those of other trials on the contralateral side. However, in cases with bilateral dominance, no M1 trials yielded “global aphasia” or “no language impairment.”

## Discussion

This study investigated the feasibility of selective anesthetic injections into the MCA, revealing the safety and efficacy of the ssWada. Additionally, it revealed a high incidence of global aphasia following anesthetic injections into the M1 of the dominant hemisphere in patients with unilateral language function.

No irreversible adverse effects were observed in the 50 trials. Behavioral abnormalities (transient confusion) were observed in two trials (4%), with no other significant adverse events affecting cognitive assessment recorded. Therefore, the incidence of adverse effects in the present study was lower than that reported for the cWada [6, 8]. Fujii et al. [1] reported that their MCA Wada test caused mild consciousness disturbance in 6 (18%) and apathy in 1 (3%) of 34 trials, in contrast to transient coma in 3 (43%) and mild consciousness impairment in 2 (29%) of 7 trials of intracarotid propofol infusions. Our results align with these results, suggesting that more selective injections can help minimize drug side effects.

Previous studies involving the cWada have often noted that some infusions into the language dominant hemisphere do not result in global aphasia, even in unilateral cases. For example, Hart et al. [2] reported that 8 of 15 patients with left dominance passed a semantic comprehension task even during left-hemispheric anesthetization. Although Wada and Kirikae [11] noted this phenomenon in their preliminary report, they did not record the rate of preserved comprehension. In a previous study, we demonstrated that localized anesthetic administration produces symptoms similar to those of various stroke-related aphasias [3]. In this study, for patients with unilateral left or right language function, anesthetic injection into the M1 caused “global aphasia” or “no language deficits” in over 95% of the trials. Notably, among the instances of “global aphasia” or “no language deficits,” there was 100% concurrence with the outcomes of other trials within each case. In the ssWada, we confirmed the area for injecting the anesthetic agent via super-selective catheterization and digital subtraction angiography. We then selectively injected the anesthetic into a restricted area, which we assumed ensured adequate perfusion of the anesthetic over language-related areas while avoiding adjacent regions. Super-selective catheterization also prevented anesthetics from affecting the contralateral hemisphere or other brain areas, which may cause unconsciousness or psychiatric symptoms, thus contributing to the clarity of the results.

Our results may also contribute to reducing the number of unnecessary injections during the selective Wada test. If a patient has unilateralized language dominance, propofol infusion into the M1 on either side would result in “global aphasia” or “no language impairment” in 95.5% of cases, as observed in 21 of the 22 cases in this study. Thus, if this strong correlation could be consistently replicated, over 95% of patients would require only one unilateral propofol perfusion to determine language dominance, leaving only those with uncertain outcomes to undergo bilateral propofol infusions.

Although our study highlights novel and significant findings that could improve patient welfare, it has some limitations. First, the sample size was relatively small, and larger studies are required to confirm our findings. Second, our study included only patients with epilepsy, limiting the generalizability of our results to other patient populations. Third, our study did not directly compare the results of the cWada with those of the ssWada. This limitation remains unaddressed due to the ethical necessity of ensuring only the minimal invasiveness required for making treatment decisions.

## Conclusion

We investigated the effectiveness and accuracy of the ssWada in determining language dominance. The test provided detailed and precise assessments of language lateralization in epilepsy patients, thus demonstrating promising results. Our findings suggest that in more than 95% of typical cases, language dominance can be determined with a single injection, making the procedure more efficient and more comfortable for the patient. While we observed a high incidence of global aphasia following anesthetic injections into the M1 of the dominant hemisphere in patients with unilateral language function, we acknowledge the need for further studies and a larger sample size to validate our findings.

## Data Availability

Not applicable.

## Abbreviations

cWada: Classical Wada test
M2inf: M2 inferior division of the middle cerebral artery
M2sup: M2 superior division of the middle cerebral artery
MCA: Middle cerebral artery
ssWada: Super-selective Wada test

## Declarations

### Previous presentations

Parts of the results from this paper were previously presented as an oral presentation, “Superiority of Super-Selective Wada Test over Classical Wada Test: Correspondence between the Dominant Hemisphere and Language Symptoms,” at the INS 2023 Taiwan meeting, and were published as an abstract. This manuscript has not been published elsewhere and is not under consideration for publication by any other journal.
